# Determination of hemodynamic risk for vascular disease in planar artery bifurcations

**DOI:** 10.1038/s41598-018-21126-1

**Published:** 2018-02-12

**Authors:** Alberto Otero-Cacho, María Aymerich, M. Teresa Flores-Arias, Miguel Abal, Ezequiel Álvarez, Vicente Pérez-Muñuzuri, Alberto P. Muñuzuri

**Affiliations:** 1 Faculty of Physics, Univ. Santiago de Compostela, 15782 Santiago de Compostela, Spain; 2 Health Research Institute of Santiago de Compostela (IDIS), Complexo Hospitalario Universitario de Santiago de Compostela (CHUS). SERGAS, Santiago de Compostela, 15706 A Coruña, Spain; 3 CIBER de Enfermedades Cardiovasculares (CIBERCV), Madrid, Spain

## Abstract

Understanding hemodynamics in blood circulation is crucial in order to unveil the mechanisms underlying the formation of stenosis and atherosclerosis. In fact, there are experimental evidences pointing out to the existence of some given vessel configurations that are more likely to develop the above mentioned pathologies. Along this manuscript, we performed an exhaustive investigation in a simplified model aiming to characterize by means of physical quantities those regions and configurations in vessel bifurcations that are more likely to develop such pathologies. The two-fold analysis is based, on the one hand, on numerical simulations (via CFD) and, on the other hand, on experiments realized in an ad-hoc designed polydimethylsiloxane (PDMS) channel with the appropriate parameters and appropriate fluid flows. The results obtained demonstrate that low velocity regions and low shear stress zones are located in the outer walls of bifurcations. In fact, we found that there is a critical range of bifurcation angles that is more likely to vascular disease than the others in correspondence with some experimental evidence. The effect of the inflow velocity on this critical range is also analyzed.

## Introduction

Cardiovascular diseases, specially of ischemic etiology, are leading causes of death in humans^1^. Atherosclerosis is a chronic and systemic inflammatory disease of the arterial vessels characterized by the formation of intimal lesions (atherosclerotic plaques) in the vasculature. Main factors interplaying in plaque formation are hemodynamic factors, biological factors, and systemic risk factors. Despite the fact that the entire arterial tree is exposed to known systemic risks factors such as hypercholesterolemia, hypertension and diabetes, atherosclerotic plaques development occurs at geometrically predisposed areas, such as in the vicinity of branch points, the outer wall of bifurcations and the inner wall of curvatures, which are sites of low shear stress, turbulence and/or oscillating flow^2–4^. Hemodynamic factors regulate multiple aspects of vascular biology and physiology and play a key role in vascular homeostasis and, as a consequence, in the development of vascular problems like atherosclerosis, aneurysms^5^ or stenosis^6^. From all the local hemodynamic forces, endothelial shear stress, the frictional force per area exerted at the vessel wall by the flowing blood, is the most widely recognized for its fundamental role in atherosclerosis^7,8^. One of the most interesting data relating low shear stress with the location of vascular lesions is the pathological observation that within a given cross section of a susceptible site, the initial lesion is almost always eccentrically distributed in accordance with the predicted low wall shear stress.

Artery bifurcation regions exhibit an inherently complex local hemodynamic microenvironment, which subsequently impacts on the localization, progression and clinical outcomes of plaque formation and particle sedimentation. After bifurcation, the velocity profile changes and a flow separation occurs after entering the daughter vessel. In flow separation, the fluid flow becomes detached from the surface of the blood vessel. The incorporation of computational fluid dynamics simulation to the analysis of such bifurcations would facilitate more precise and clinically relevant assessment of geometry and wall shear stress in artery bifurcations, leading to better clinical outcomes. It is difficult to say if hemodynamic parameters are themselves causative of the disease, but they “prime the soil” in which lesions develop, interacting with the other factors involved^9^. The study of fluid flow of the vascular system requires a combination of numerical and *in vivo* and *in vitro* experimental studies. *In vivo* studies are only possible in very limited situations due to practical and ethical reasons. Numerical studies always require experimental validation due to the importance of turbulence, non-Newtonian effects and the particulate nature of blood. Our aim was to combine the numerical study with its experimental validation in order to define the predictable hemodynamic risk of planar arterial bifurcations.

It is well known that coronary circulation consists of two to three major branches. The right side of the heart is fed by the right coronary artery (RCA) whereas the left side is fed by (1) the left main coronary artery (LM), which bifurcates into the left anterior descending artery (LAD) and the left circumflex (LCx), or (2) The LAD and LCx when they stem directly from the aorta. Of all bifurcations in the coronary circulation, LM-LAD-LCx bifurcations are most often affected by disease^10^. Most of the percutaneous coronary interventions affect the bifurcation of the LM into the two branches (LCx and LAD). In fact, there is a large variety of angles characterizing this bifurcation and its importance has been analyzed in previous works^11,12^.

In this paper we consider the idealized version of the bifurcation described above and analyze the effect of the physical parameters aiming to characterize the areas within the vessel that are more likely to develop pathologies. We propose to characterize these areas as the regions where anomalies in the normal flow circulation cause the appearance of low velocity areas. Here the flow remains during larger periods of time and thus (1) pathological particles have more time to deposit and bound to the vessel boundary, and (2) low shear stress at the endothelium of these zones make this “soil” more prone to disease.

The paper is organized as follows. First, we present the results obtained both in numerical simulations and experiments (details of the models and protocol followed are described in the Supplementary Information). The paper is concluded with some discussion of the results and conclusions.

## Results

**Figure 1:**
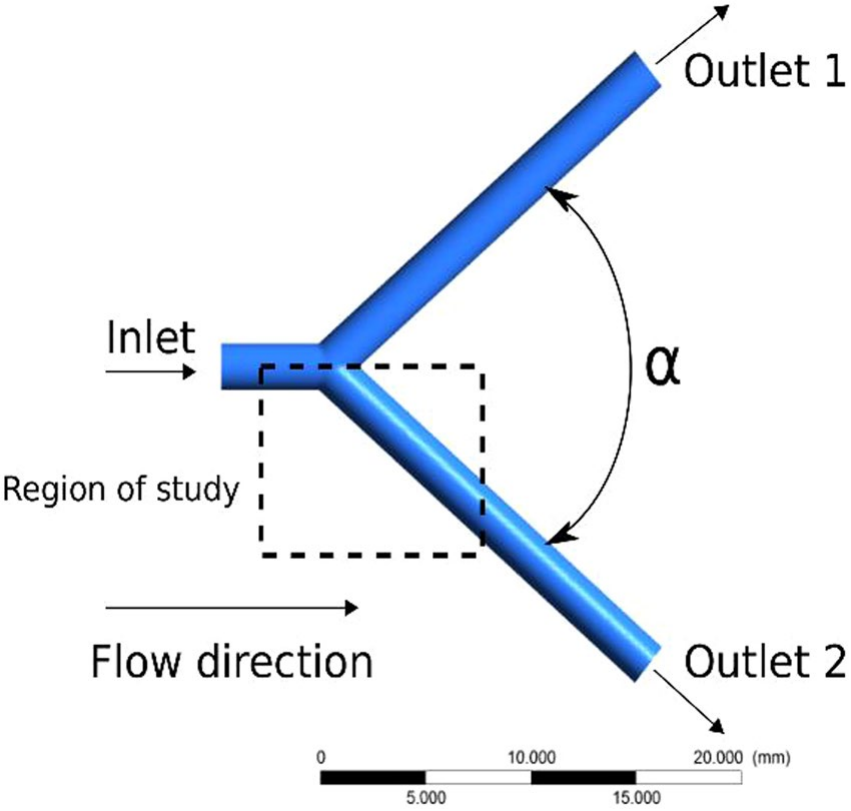
General Scheme. Bifurcation of a mother blood vessel into two identical daughter vessels.

Blood circulation in a coronary artery bifurcation is numerically simulated following the description in the Supplementary Information (SI). The geometry considered is an idealization of the LM bifurcation into the two branches (LCx and LAD). It consists in a main vessel that splits into two after a bifurcation point is reached. The control parameter is the bifurcation angle. Figure 1 presents a scheme with the main features of this configuration.

In experiments, PDMS semicircular channels are made as described in the Supplementary Information (SI) to analyze circulation. Images are taken with a CCD camera and further image processing allows us to extract information about the flow velocities at each location of the channel.

**Figure 2:**
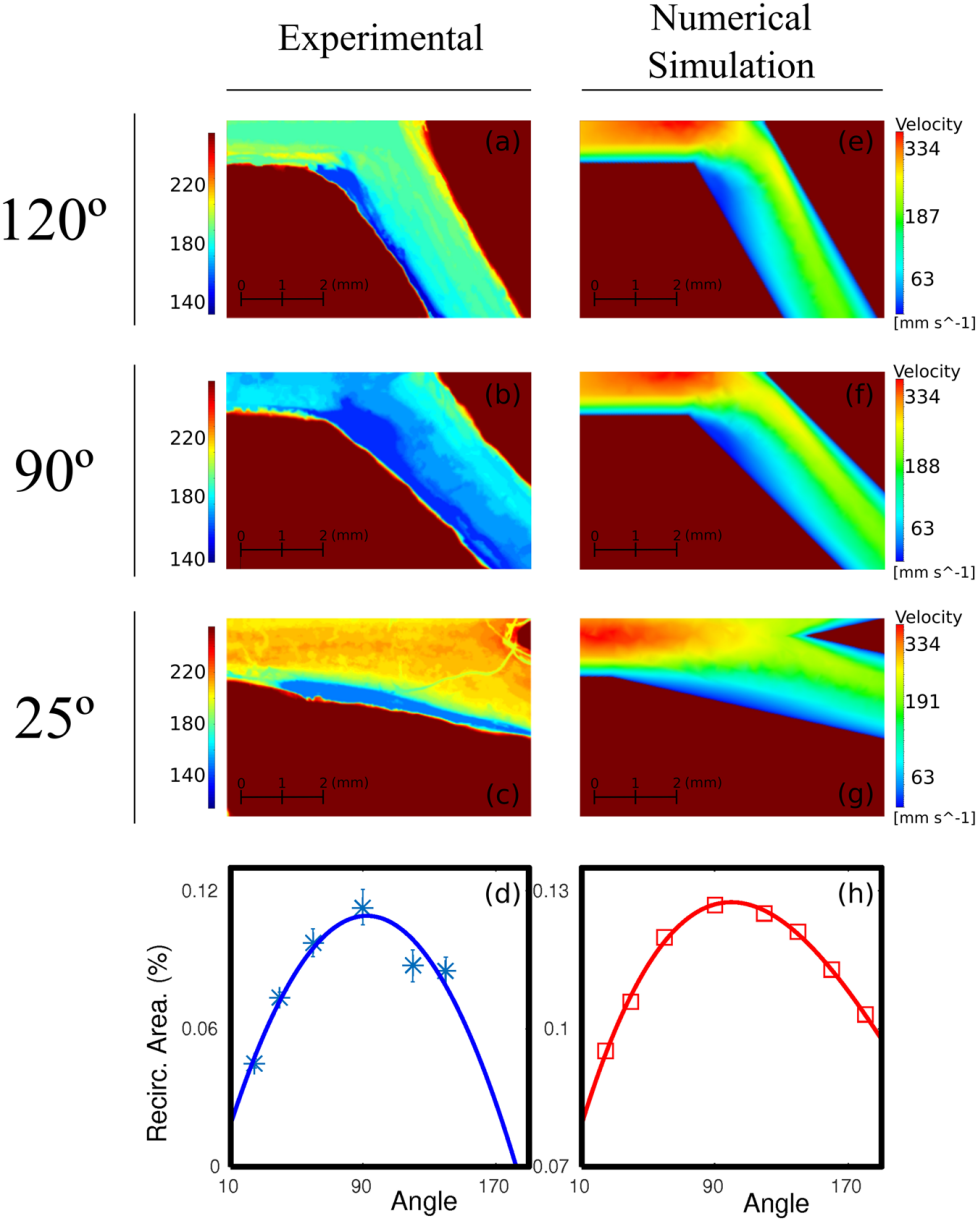
Comparison between experimental and numerical simulation data in half geometry for three different bifurcation angles (25°, 90° and 120°). First column (panels a to c) present (color coded) the spatial distribution of the flow velocity in the channel when a sucrose solution is circulated in the device at 27 ml/min. The column on the right shows the flow velocity magnitude considering an input velocity of 0.2 m/s. The sizes of the areas of low circulation are plotted in panels (d) for experiments and (h) for numerical simulations for different bifurcation angles.

A comparison of experimental and numerically computed results is plotted in Fig. 2. Left column (panels a to c) shows (color coded) the spatial distribution of the ferroin solution during the discharge period at each location of the experimental channel for three different bifurcation angles. Dark blue areas correspond to a higher concentration of colorant and it is related with lower values of flow velocity and, thus, with regions where potentially pathological particles are more likely to deposit. Areas in red correspond with the extra vessel space. The liquid always flows from left to right and only the lower branch after the bifurcation is presented (the other exhibiting the symmetrical behavior as expected). The regions with lowest flow velocities are always located next to the external wall of the bifurcation. The flow separates after the bifurcation and a low flow velocity area is created in the separation area. Note that the largest region in dark blue happens for the intermediate angle of 90°.

The corresponding numerical simulations are plotted on the right column (panels e to g). Here, velocity values inside the channel are represented and, again, regions in dark blue color correspond with low velocity. A similar behavior as the one observed in experiments is shown.

A summary of all the results including numerous additional experiments is plotted in the lower two panels (left, panel d, for experiments and right, panel h, for numerical simulations). Here the low velocity area is plotted versus the bifurcation angle. Each value was averaged over at least 5 realizations. The low velocity area is measured as the region where circulation flow decays below a certain arbitrary threshold and normalized by the observation area (see appendix for details). In these two panels we see that there is an angle, around 90°, for which the region of low flow velocity reaches a maximum. The region where the flow slows down is significantly larger for this critical angle and thus the possibilities for a pathogen particle to stablish a connection with the boundary are increased. Note that the actual values of the low velocity areas are not the same for the experimental and numerical cases. This is due to the different methods used to determine these areas as described in the methods section.

**Figure 3:**
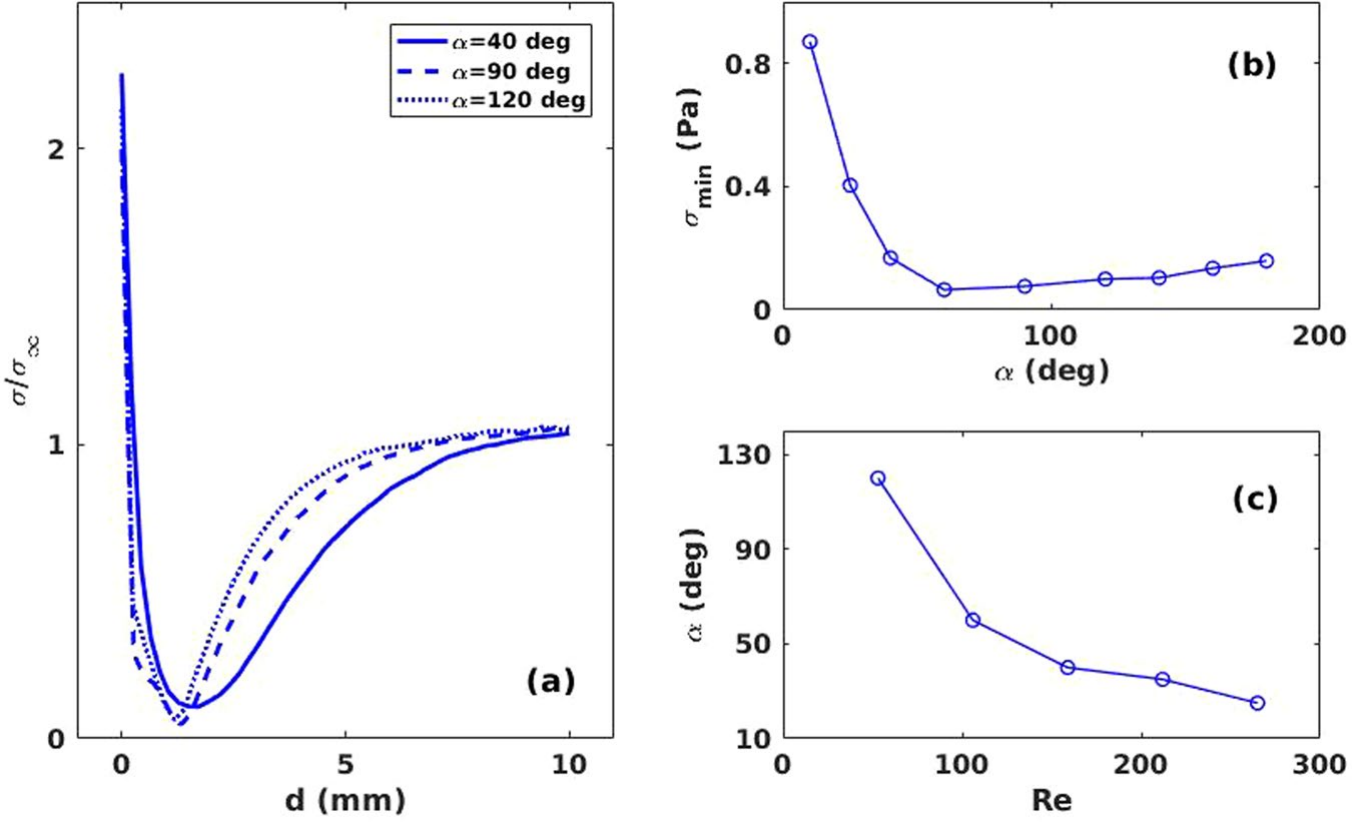
(**a**) Wall shear stress versus distance to the bifurcation along the outer bifurcation wall for different bifurcation angles. σ_∞_ = 1.55 Pa (**b**) Minimum wall shear stress as a function of α. (**c**) Bifurcation angle in which the lower value of wall shear stress appears versus Reynolds number.

In the numerical simulations, a complete description of the flow in the channels is obtained with the required spatial resolution. Wall shear stress σ is a widely used parameter to determine which zones are more prone to form plaque. This magnitude accounts for the frictional forces between the circulation flow and the vessel walls and it becomes zero when the flow stops. Blood vessel walls with low shear stress are more likely to deposit plaque and thus develop atherosclerosis^7,8^. Figure 3a shows the variation of the wall shear stress along the outer bifurcation wall measured as a function of the longitudinal distance from the bifurcation point for different bifurcation angles. Figure 3b shows the minimum of the WSS versus the channel angle. Note that the lowest value of the wall shear stress is attained for α = 60°. As the flow conditions are changed, this critical angle is also changed. The results are plotted in Fig. 3c. Here it is possible to see that as the Reynolds number increases the critical angle becomes smaller, thus, for high velocities (as in the coronary arteries), acute angles are more prone to plaque formation.

In order to check the robustness of our results, simulations with a pulsatile flow were also performed. The results (shown in the Supplementary Information, section 3) are in agreement with those presented here, i.e., there is a critical angle at which plaque formation is favored.

Another way to determine the regions where plaque is more likely to appear is analyzing the critical bifurcation angle needed to observe a recirculation area. The results obtained with this analysis (shown in the Supplementary Information, section 2) are consistent with those here shown.

## Discussion and Conclusions

We constructed an *in-vitro* idealization of a vessel bifurcation made with PDMS and made a sucrose flow circulate through it mimicking the actual blood circulation in a living being. A mathematical model of this process was also developed and the simulated results coincide and complement those from experiments.

An exhaustive investigation of both systems allowed us to determine the low flow velocity areas. Both sources of information yield to equivalent results. They demonstrate that low velocity zones and low shear stress areas are located in the outer walls of a bifurcation (symmetric Y-junction). Furthermore, it was found that there is a critical range of bifurcation angles that could be more prone to vascular disease and particle adhesion than the others. In some studies, the incidence of the bifurcation angle in plaque formation has been studied in *in-vitro*-experimental models reaching very similar conclusions^13^. However, although a wide consensus is found on the location of low wall shear stress in the outer walls of the bifurcation, the effect of the bifurcation angle is still unclear. Chiastra *et al*.^14^ recently analyzed the influence of the bifurcation angle and cardiac curvature of idealized models of stenosed and unstenosed bifurcations of the LAD coronary artery with its diagonal branch. Their computational fluid dynamics simulations concluded that the bifurcation angle had a minor effect on the local hemodynamics. However, they limited the analysis to the narrowed range of angles exhibited by this particular coronary bifurcation (from 40° to 70°), which only covers a part of our range for a general model and it does not include the critical angle suggested by our data. A study of a wide range of angulations (15° to 120°) on simulated models of left coronary artery came to the conclusion that disturbed flow pattern and low wall shear stress was observed in the models with wider angulations (110º–120°)^15^, which differ from our finding that the worse flow conditions are achieved for a range of angles in the interval [60°, 90°].

Dong *et al*.^16^ analyzed the branch angulation of the LM in simulated models under pulsatile blood pressure. They concluded that high tensile and low oscillatory shear stress simultaneously occurs at the branch side bifurcation shoulder in wider-angled models. In a pulsatile flow regime, the actual measured parameters may change with time, but still the low velocity areas develop after the bifurcation and in the outer walls. The simulation of compliant vessels, as well as the consideration of Newtonian and non-Newtonian flows, seems to be important for the estimation of the size and even for the localization of low wall shear stress^17^.

In conclusion, our results suggest that low flow velocity zones and low shear stress values are located in the outer walls of all the bifurcation angles. For the particular case of a velocity value equal to 0.2 m/s it was found that there is a critical bifurcation angle close to the range [60°, 90°] that it is more likely to vascular stenosis and particle adhesion. Complementary results shown in Supplementary Information considering a pulsatile flow and different inflows support the generality of the results here presented. Even more, increasing the inflow velocity is shown to shift the critical angle range into smaller values and more acute angles. Our conclusions can also be of relevance understanding the migration of metastasizing cancer cells through the blood stream and the eventual development of metastasis.

## Methods and Materials

### Numerical Model

Star-CCM+ software^18^ was used to design the geometries, convert this geometries into a grid and carry out numerical simulations solving the fluid-dynamics equations using finite volume method^19,20^. Segregated Flow solver (according to the SIMPLE algorithm) was used. Blood dynamic has been simulated by the three-dimensional, incompressible Navier-Stokes (NS) equations. Reynolds numbers smaller than 200 were considered for each configuration (thus laminar flow is always granted). At the same time, circulating blood was considered as a Newtonian fluid with no-slip condition at the vessel rigid walls. In order to solve the NS equations, a 3D mesh was designed taking into account that most of the important recirculation zones are located close to the outer bifurcation vessel walls^2^. The mesh is composed of tetrahedral and rectangular prisms being more accurate near the walls in order to study the low velocity zones with more precision (see Supplementary Information, section 4). The convergence criterion of reduction of residuals by five orders of magnitude was used. Computations were run until a steady state was reached.

Eight different configurations of symmetric Y-junction vessels were studied with angles ranging from 25° up to 180°. For the simulations here presented, an inlet velocity condition consisting in a flat profile of 0.2 m/s^21^ was considered in the main vessel and atmospheric pressure outlet in both branching vessels, except specified otherwise. Although it is well known that there are other outflow conditions more suitable to correlate simulations with real circulatory system (such as Murray law-based conditions) an atmospheric pressure condition was used as a first approximation to the problem in order to compare the experimental data. All vessels have identical cross-sectional area D = 2 mm. The density of the circulating fluid was set ρ = 1060 kg/m^3^ and the viscosity μ = 0.004 kg/(m s).

Each simulation provides a complete spatial description of the flow velocities. These values were directly plotted in Fig. 2 color coded.

In order to study the low velocity zones it was necessary to establish a velocity value below which a given zone is considered as a low velocity zone. Zones with velocity values less than a 10% of the maximum velocity recorded were considered low velocity areas. To carry out the study the velocity module for the z = 0 plane section was represented. These low flow areas were measured considering a length of 5 mm on the outer walls of the branches from the bifurcation point in order to eliminate from the calculations, as far as possible, those areas with low velocities near the wall that are not of interest for our analysis. Results were also complemented with simulations considering a pulsatile flow as an inlet condition (see Supplementary Information, section 3).

In addition, the recirculation zones have also been studied considering negative flow velocity in relation to the main flow direction^22^.

### Experimental Model

Six different PDMS semicircular channels were made (with angles in the range [25°, 140°]) to experimentally analyze circulation. PDMS is a biocompatible material widely employed in the fabrication of biological devices. It presents advantageous properties for bioassays, such as transparency, permeability to gasses, accuracy in the replica until nanometer and chemical inertness^23^. Due to its properties, it has been the material selected for the fabrication of the channel in this work. PDMS is presented as a kit of monomer and hardener where the ratio between them can be modified in order to obtain results with different elastic properties. In this case, we have mixed PDMS in a ratio 20:1 and 5:1 for the cover and the channel, respectively. This is not a completely rigid device. However, because the channel through which the flow passes is embedded in a PDMS structure that limits the movements of the walls and taking into account the range of pressures that are reached inside the channel, it seems appropriate to assume the consideration of rigid walls (condition also used in numerical simulations). The fabrication of the device that was used in the experimental validation of the simulations is described in^24^. Briefly, laser technologies were used for manufacturing the channel over soda-lime glass. A Nd:YVO_4_ Rofin laser with 1064 nm wavelength and 20 ns pulse duration was focused over a metal foil placed below the soda-lime substrate. The millimeter dimension channel was fabricated by using laser backwriting technique^25^ and laser parameters were 8 W average power, 1000 mm/s scan speed and 12 kHz repetition rate. A post-thermal treatment was applied to the soda-lime glass master in order to enhance its quality. The sample was heated into a static oven at 590 °C, which is above the transition temperature of the soda-lime glass, for two hours. Finally, the master was replicated in polydimethylsiloxane (PDMS) by well-known soft-lithography methods^26^. In order to enclose the channel and to obtain an irreversible bonding between the structure and a PDMS cover, Unger *et al*.^27^ techniques were used. By the described method, a device in PDMS that mimics a coronary blood vessel was achieved.

**Figure 4:**
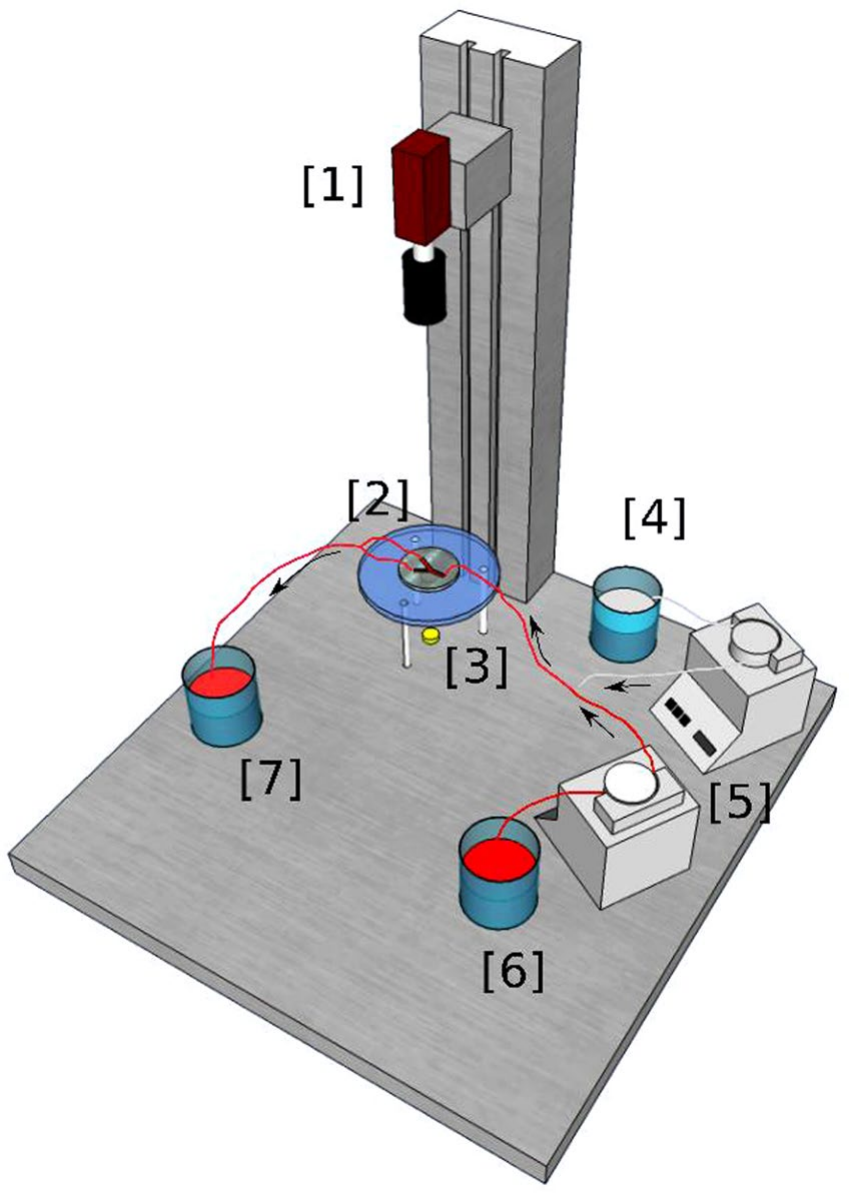
General scheme of the experimental setup. Two peristaltic pumps are used to pump ferroin and sucrose solution from their corresponding reservoirs. The mixture of both fluids occurs in a Y-shaped bifurcation and after that, the mixture enters the PDMS geometry that is illuminated with a LED. There, the flow is registered by a CCD camera. Finally, the mixture exits to a third reservoir. [1] CCD Camera, [2] PDMS Geometry, [3] LED, [4] Reservoir (Sucrose Solution), [5] Peristaltic Pumps, [6] Reservoir (Ferroin), [7] Reservoir (Ferroin + Sucrose Solution).

Once the channel device was produced, it was incorporated into the experimental setup depicted in Fig. 4. A sucrose solution (35%) (with values of viscosity and density similar to those of blood, see Table 1) was circulated through the device. The solution was stored in a container and pumped through the system with a peristaltic pump (GILSON, Minipuls 3 Peristaltic Pump). The two outputs of the device were let open at the atmosphere in order to compare with the simulations. The device was illuminated from below and a CCD camera was placed above to record images.

**Table 1:** Fluid characteristics adopted for experimental and numerical analysis and blood characteristics in human circulatory system^28,29^.

	Density (g/cm^3^)	Viscosity (mPa s)
Sucrose solution	1.127	4.16
Numerical simulation	1.060	4
Blood in human circulatory system	1.060	4

The low velocity areas are determined following the protocol described in the Supplementary information using technics that minimize the interference with the flow.

## Electronic supplementary material

Supplementary Information

Low velocity areas determination, bifurcation angle 25º

Low velocity areas determination, bifurcation angle 60º
