# Supplementary material for: Determination of hemodynamic risk for vascular disease in planar artery bifurcations
Source: Sci Rep. 2018 Feb 12;8:2795. doi: 10.1038/s41598-018-21126-1 (PMC5809427; doi:10.1038/s41598-018-21126-1)
Supplement: Supplementary file 1 — Supplementary Information [file 41598_2018_21126_MOESM1_ESM.doc]

| SUPPLEMENTARY INFORMATION |
| --- |

Determination of hemodynamic risk for vascular disease in planar artery bifurcations

Alberto Otero-Cacho[[1]](#footnote-2), María Aymerich^1^, M.Teresa Flores-Arias^1^, Miguel Abal[[2]](#footnote-3), Ezequiel Álvarez^2^, Vicente Pérez-Muñuzuri^1^ and Alberto P. Muñuzuri^1*^

Index:

1. Experimental analysis
2. Recirculation areas
3. Pulsatile Flow
4. Computational grid

**1. Experimental analysis**

In order to determine the the low velocity areas in the medium (plotted in Fig. 2) the following protocol is considered. Two different solutions are pumped through the device. One containing only the sucrose solution (35%) as described before that it is constantly pumped with a flow rate of 27 ml/min. Images taken under these conditions are transparent and the flow velocities cannot be characterized. In order to signal the differential velocities in the flow, we additionally inject a second solution (during 10 sec. at a flow rate of 2.5 ml/min) containing a colorant (Ferroin 25 mM). This gradually changes the color of the solution till a homogeneous red.

The evolution of the average intensity with time is plotted in Fig. SI-1. The sequence of images considered here starts when the colorant begins to be pumped into the system and, thus, the transmitted intensity reaching the camera is decreased. Note that after some time, when the colorant starts to clear out, the light intensity reaching the camera starts to increase till the normal values are achieved again (see movies 25g.mp4 and 60g.mp4). All frames located in section ‘Ferroin discharge’ (Fig. SI-1) are added pixel by pixel and calculate the average intensity at every point. The resulting images are shown in Fig. 2. Note that those areas with lower velocities need more time to clear all the colorant off and thus the final value after the summation is slightly darker.


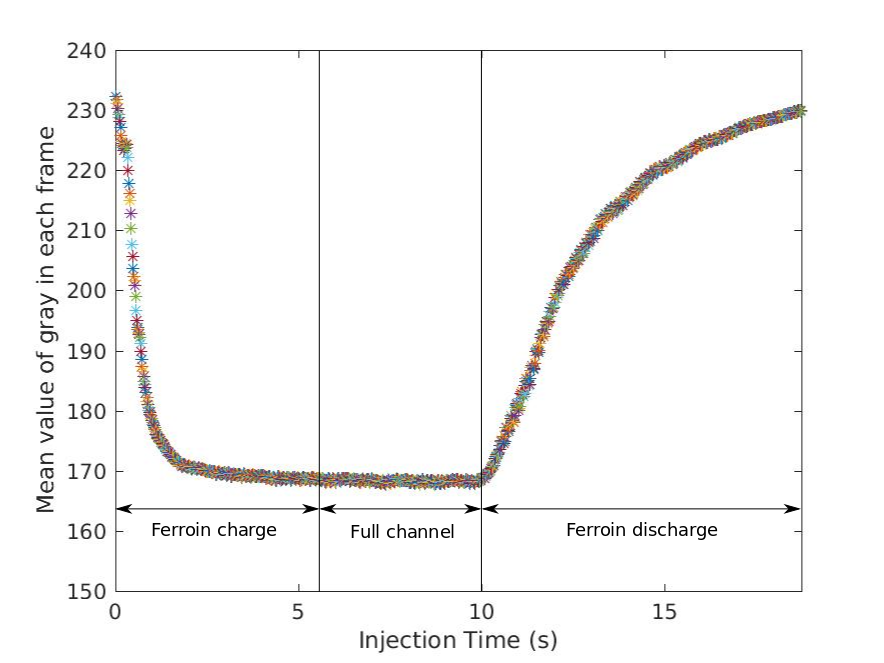


Figure SI-1. Colorant evolution within the geometry. Evolution of mean value of gray in each frame considered within the time.

Another example is plotted in Fig. SI-2. Fig. SI-2A plots the result of adding all images taken during the Ferroin discharge period. Fig. SI-2B is just a contour map of the previous plot clearly showing the low velocity area. Note that this method is less intrusive and thus interferes less with the flow.


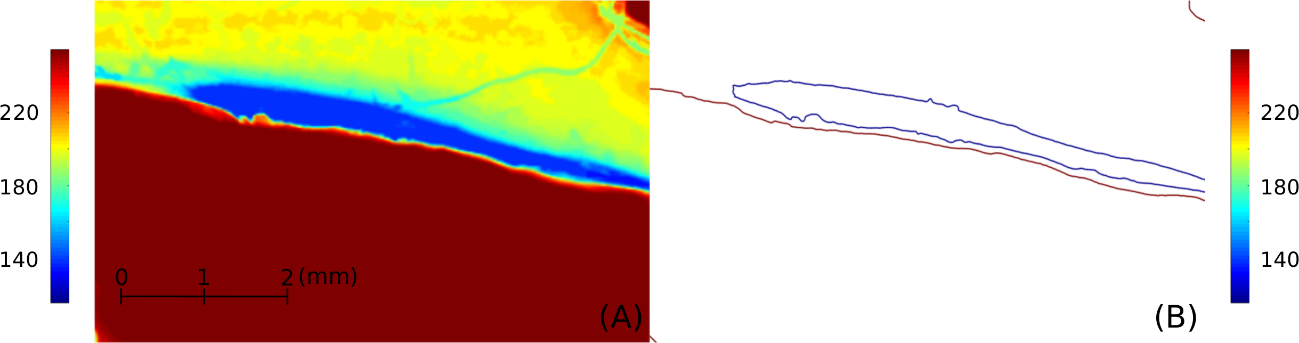


Figure SI-2. (A) Represent, color coded, the spatial distribution of the ferroin solution during discharge period in the channel (25°). (B) Representation of the wall contour (red) and 160 gray contour (blue). Area inside blue line would be the area used to characterize low velocity area.

**2. Recirculation areas**

Using a similar method to that used by Martorell et al. (2014) to determine de presence of a recirculation area, velocity direction was analyzed and compared with the direction given by the vector that defines the arterial branch (shown in Fig. SI-3). If the velocity is negative, we considered that a recirculation zone exists.


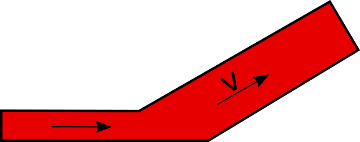


Figure SI-3. Representation of positive velocity vector

With the considerations made above, the occurrence of recirculation zones was studied for several velocity inlet values and results presented in Fig. SI-4.


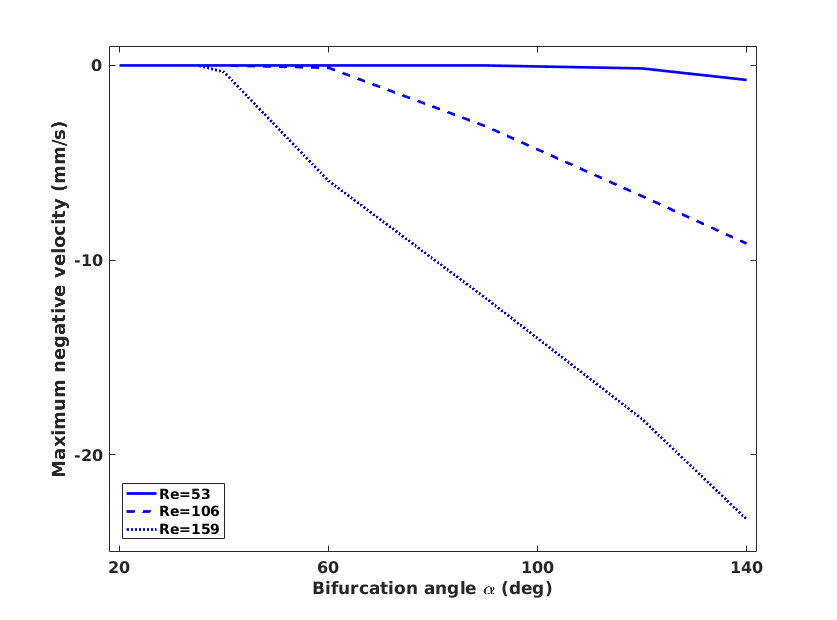


Figure SI-4. Maximum negative velocity for different bifurcation angles for different velocity inlet values (0.1m/s, 0.2 m/s, 0.3 m/s).

The angle of occurrence of the recirculation zones (when the negative velocity appears for the first time) depending on the velocity inlet is plotted here. It is in good agreement with the angle at which the lowest value of shear stress occurs. Note that the angle of occurrence of the recirculation is lower as the inlet velocity increases.

**3. Pulsatile Flow**

Pulsatile flow was included in the CFD simulation by imposing a time-varying velocity boundary condition at the entrance of the artery and considering atmospheric pressure for outlets. Blood flow wave form and velocities were estimated from previously published data [23] and the period used was 0.81 s corresponding to a heartbeat of 74 beats per minute.

The Womersley number is a dimensionless parameter used to characterize pulsatile blood flow in arteries. It is the ratio of inertial forces relative to viscous forces and its expression is given by:


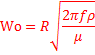


where R is the radius of the artery, f is the pulsatile frequency,  *ρ* is the fluid density and *μ* is dynamic viscosity.

For the pulse described above, where the arterial diameter is 0.002m, Wo=1.434.

The evolution of WSS depending on pulse time is shown in Figure SI-5. Note that the behavior of WSS with alpha for P1 and P2 positions is the same as for a regular constant flow inlet.


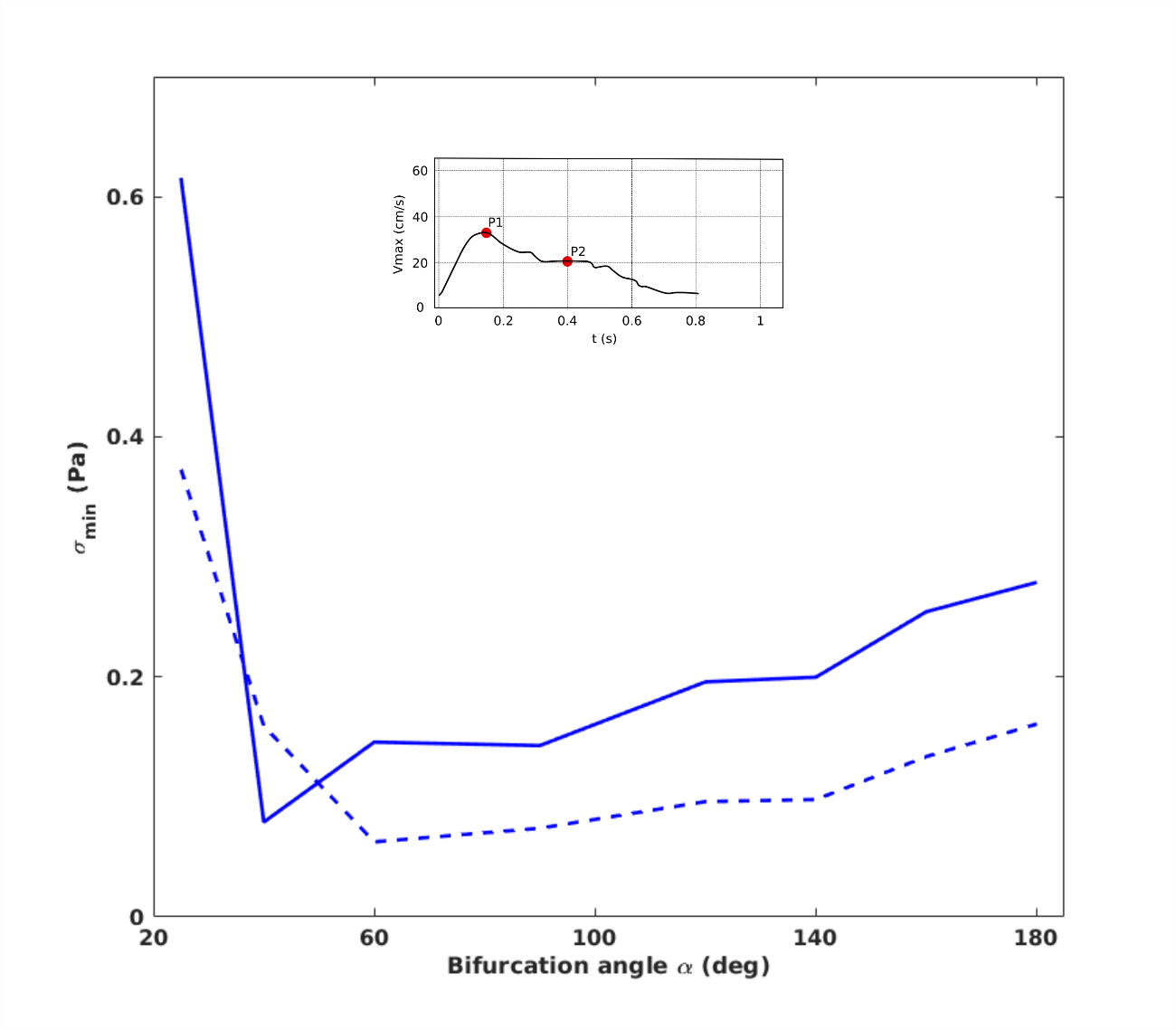


Figure SI-5. Minimum WSS as a function of the bifurcation angle for two instants of the pulse sequence.

**4. Computational Grid**

Star-CCM+ allows the creation of a tetrahedral mesh refined near the walls with hexahedral layers where grids area clustered in radial direction to improve the accuracy in regions with high velocity gradients. Three grids of different grid density and size were considered with 144269, 257733 and 529481 elements. All the simulation cases were repeated for Grid 1, Grid 2 and Grid 3. Maximum difference in the values of maximum velocity and low flow area between all the grids considered here was less than 5%.

Details of the grid analysis for 60° angle can be found on the table SI-6:

| 60° geometry | | | | | |
| --- | --- | --- | --- | --- | --- |
|  |  | Parameters | | Variations | |
|  | Number of elements | Max. velocity | Low flow regions (area) (mm2) | Max. velocity | Low velocity flow regions |
| Grid 1 | 144269 | 0.3431704 | 1.202238 | - | - |
| Grid 2 | 257733 | 0.346058 | 1.222501 | 0.83% | 1.66% |
| Grid 3 | 529481 | 0.3474783 | 1.247171 | 1.24% | 3.60% |

Table SI-1: Details for different grids

Due to the small differences observed in the obtained results, we opted to perform all the calculations using the grid 1 configuration in order to optimize the computer time usage.

1. Faculty of Physics. Univ. Santiago de Compostela. 15706 Santiago de Compostela. Spain [↑](#footnote-ref-2)
2. Health Research Institute of Santiago de Compostela (IDIS); Complexo Hospitalario Universitario de Santiago de Compostela (CHUS). SERGAS. Santiago de Compostela, 15706 A Coruña, Spain, and CIBER de Enfermedades Cardiovasculares (CIBERCV), Madrid, Spain. [↑](#footnote-ref-3)
